# Role of Transmitted Gag CTL Polymorphisms in Defining Replicative Capacity and Early HIV-1 Pathogenesis

**DOI:** 10.1371/journal.ppat.1003041

**Published:** 2012-11-29

**Authors:** Jessica L. Prince, Daniel T. Claiborne, Jonathan M. Carlson, Malinda Schaefer, Tianwei Yu, Shabir Lahki, Heather A. Prentice, Ling Yue, Sundaram A. Vishwanathan, William Kilembe, Paul Goepfert, Matthew A. Price, Jill Gilmour, Joseph Mulenga, Paul Farmer, Cynthia A. Derdeyn, Jiaming Tang, David Heckerman, Richard A. Kaslow, Susan A. Allen, Eric Hunter

**Affiliations:** 1 Emory Vaccine Center at Yerkes National Primate Research Center, Emory University, Atlanta, Georgia, United States of America; 2 Microsoft Research, Los Angeles, California, United States of America; 3 Department of Biostatistics and Bioinformatics, Emory University, Atlanta, Georgia, United States of America; 4 Zambia-Emory HIV Research Project, Lusaka, Zambia; 5 Department of Epidemiology, University of Alabama, Birmingham, Alabama, United States of America; 6 Department of Medicine, University of Alabama, Birmingham, Alabama, United States of America; 7 International AIDS Vaccine Initiative, San Francisco, California, United States of America; 8 International AIDS Vaccine Initiative, London, England; 9 Department of Pathology and Laboratory Medicine, Emory University, Atlanta, Georgia, United States of America; 10 Department of Global Health, Rollins School of Public Health, Emory University, Atlanta, Georgia, United States of America; SAIC-Frederick, United States of America

## Abstract

Initial studies of 88 transmission pairs in the Zambia Emory HIV Research Project cohort demonstrated that the number of transmitted HLA-B associated polymorphisms in Gag, but not Nef, was negatively correlated to set point viral load (VL) in the newly infected partners. These results suggested that accumulation of CTL escape mutations in Gag might attenuate viral replication and provide a clinical benefit during early stages of infection. Using a novel approach, we have cloned *gag* sequences isolated from the earliest seroconversion plasma sample from the acutely infected recipient of 149 epidemiologically linked Zambian transmission pairs into a primary isolate, subtype C proviral vector, MJ4. We determined the replicative capacity (RC) of these Gag-MJ4 chimeras by infecting the GXR25 cell line and quantifying virion production in supernatants via a radiolabeled reverse transcriptase assay. We observed a statistically significant positive correlation between RC conferred by the transmitted Gag sequence and set point VL in newly infected individuals (p = 0.02). Furthermore, the RC of Gag-MJ4 chimeras also correlated with the VL of chronically infected donors near the estimated date of infection (p = 0.01), demonstrating that virus replication contributes to VL in both acute and chronic infection. These studies also allowed for the elucidation of novel sites in Gag associated with changes in RC, where rare mutations had the greatest effect on fitness. Although we observed both advantageous and deleterious rare mutations, the latter could point to vulnerable targets in the HIV-1 genome. Importantly, RC correlated significantly (p = 0.029) with the rate of CD4+ T cell decline over the first 3 years of infection in a manner that is partially independent of VL, suggesting that the replication capacity of HIV-1 during the earliest stages of infection is a determinant of pathogenesis beyond what might be expected based on set point VL alone.

## Author Summary

In the majority of HIV-1 cases, a single virus establishes infection. However, mutations in the viral genome accumulate over time in order to avoid recognition by the host immune response. Certain mutations in the main structural protein, Gag, driven by cytotoxic T lymphocytes are detrimental to viral replication, and we showed previously that, upon transmission, viruses with higher numbers of escape mutations in Gag were associated with lower early set point viral loads. We hypothesized that this could be attributed to attenuation of the transmitted virus. Here, we have cloned the *gag* gene from 149 newly infected individuals from linked transmission pairs into a clade C proviral vector and determined the replicative capacity *in vitro*. We found that the replicative capacity conferred by the transmitted Gag correlated with set point viral loads in newly infected individuals, as well as with the viral load of the transmitting partner, and we identified previously unrecognized residues associated with increasing and decreasing replicative capacity. Importantly, we demonstrate that transmitted viruses with high replicative capacity cause more rapid CD4+ decline over the first three years, independent of viral load. This suggests that the trajectory of pathogenesis may be affected very early in infection, before adaptive immunity can respond.

## Introduction

Despite a diverse HIV-1 quasispecies within chronically infected individuals, a single variant establishes infection in the majority of heterosexual transmission cases, resulting in a severe genetic bottleneck [1]–[3]. A more profound understanding of the interaction between host and viral characteristics and how they shape early pathogenesis and disease progression will be integral for understanding the trajectory and impact of early events after heterosexual transmission. While it is well established that host factors such as HLA-class I alleles can play a major role in determining clinical progression in those individuals recently infected with HIV-1 [4]–[10] the role of transmitted viral characteristics has been understudied due to the lack of suitable cohorts in which virus from both the donor and linked recipient are available. Accordingly, studies using epidemiologically linked heterosexual transmission pairs are essential for understanding how viral evolution, adaptation, and the characteristics of the transmitted variant influence HIV-1 pathogenesis.

Previous studies in both heterosexual and homosexual transmission pairs have demonstrated that viral loads (VL) in the newly infected partners are correlated with VL in the transmitting partner [11]–[13]. This finding is intriguing as the majority of the linked couples have disparate HLA-I alleles, and would therefore have varying immune responses to the incoming virus. Thus, the characteristics of the virus in the donor quasispecies that impact replication can similarly impact the replication of the newly infecting virus even in the context of what is frequently a distinct immunogenetic environment. Indeed, when host factors known to modulate VL such as the presence of protective or unfavorable HLA-I alleles, gender, age, and HLA-B sharing are taken into account in a multivariable analysis utilizing a general linearized model, the correlation between donor and recipient VL becomes highly significant (Yue *et al.*, manuscript in submission).

It is clear that both humoral [14] and cellular immune responses can drive virus evolution over the course of infection. In the case of the latter, escape mutations arise that abrogate the ability of cytotoxic T lymphocytes (CTL) to kill virus-infected cells [15]–[22]. While the selection of CTL escape mutations provides an *in vivo* advantage for the virus, if a mutation occurs within a functionally constrained region of the genome such as Gag, it could reduce *in vitro* replicative fitness [17], [23]–[30]. This phenomenon has been demonstrated for several CTL escape mutations associated with protective alleles such as HLA-B*57, B*5801, B*27, and B*81 [31]–[36]. The ability of protective alleles to target conserved regions of the genome that escape with difficulty, due to the fitness costs incurred by mutations at these epitopes, may partially explain the mechanism of enhanced protection from disease progression in individuals with these alleles [37]–[41]. While evasion from the CTL response may result in such deleterious mutations, the *in vivo* fitness benefit outweighs that of the replication cost [42], and the ongoing selection of additional mutations may allow the virus to compensate for these defects [17], [29], [32], [33], [35], [43]–[49]. The functional consequence of escape and compensatory mutations upon transmission to an HLA-mismatched individual has not been fully explored.

Initial studies in the ZEHRP cohort of 88 transmission pairs demonstrated that the number of transmitted HLA-B associated polymorphisms in Gag, but not Nef, was negatively correlated to set point VL in the newly infected partners, suggesting that an accumulation of escape mutations might attenuate viral replication and provide a clinical benefit during early stages of infection [50]. In addition, in a smaller study of nine newly infected individuals infected by viruses with fitness reducing HLA-B*5703 associated epitope-escape mutations in p24, a lower set point VL was observed [24]. Previous studies have also shown that elite controllers can harbor viruses with *gag-pro* sequences that confer reduced *in vitro* replicative capacities (RC) [51]–[53]. In a series of studies, Brockman and colleagues have shown that *in vitro* RC conferred by *gag-pro* variants isolated from both subtype B and C chronically infected individuals correlates to VL, demonstrating the role of intrinsic viral characteristics in defining this marker of pathogenesis [43], [47], [54], [55]. However, in these studies the recombination of population-based PCR amplified sequences into the lab adapted NL4.3 provirus required the outgrowth of virus in a CEM-CCR5 based cell line, potentially skewing the nature of the virus recovered. In contrast, studies of HIV-1 fitness in acute infection did not yield a statistically significant correlation between RC and VL, potentially due to small sample sizes and the limitations of the methodologies used.

The identification of 149 heterosexual epidemiologically linked transmission pairs from a discordant couple cohort in Lusaka, Zambia, provides a unique opportunity to investigate the role that HLA-mediated adaptation of Gag within a chronically infected individual plays in modulating the RC of the transmitted variant. We hypothesize that HLA-mediated adaptation of HIV-1 resulting in Gag variants conferring varying levels of RCs will be a major viral characteristic linking donor and recipient VLs, and that the *in vitro* RC conferred by the transmitted Gag sequence defines early clinical parameters of HIV-1 pathogenesis.

To test this hypothesis and using a novel approach, we cloned *gag* sequences from the earliest seroconversion plasma sample from 149 newly infected recipients of epidemiologically linked Zambian transmission pairs into the MJ4 proviral backbone [56]. The RC of each Gag-MJ4 chimera was then determined and used to investigate how the RC conferred by the transmitted Gag sequence defines clinical parameters, such as early set point VL and CD4+ T cell decline in the newly infected individuals. These studies allowed us to identify novel residues in Gag that influence RC, and demonstrate a strong correlation between RC and early set point VL, as well as between RC and CD4 decline during the first three years of infection, which was also found to be independent of VL. Thus, the RC of the transmitted virus as defined by its *gag* gene influences critical aspects of HIV-1 pathogenesis.

## Materials and Methods

### Study subjects

All participants in the Zambia Emory HIV Research Project (ZEHRP) discordant couples cohort in Lusaka, Zambia were enrolled in human subjects protocols approved by both the University of Zambia Research Ethics Committee and the Emory University Institutional Review Board. Prior to enrollment, individuals received counseling and signed a written informed consent form agreeing to participate. The subjects selected from the cohort were initially HIV-1 serodiscordant partners in cohabiting heterosexual couples with subsequent intracouple (epidemiologically linked) HIV-1 transmission [57]–[59]. Epidemiological linkage was defined by phylogenetic analyses of HIV-1 *gp41* sequences from both partners [60]. Viral isolates from each partner in the transmission pair were closely related, with median and maximum nucleotide substitution rates of 1.5 and 4.0%, respectively. In contrast, median nucleotide substitution rate for unlinked HIV-1 C viruses from the Zambian cohort and elsewhere was 8.8% [60]. The algorithm used to determine the estimated date of infection (EDI) was previously described by Haaland *et al.* [2]. All patients in this cohort were antiretroviral therapy naïve. Zambian linked recipients were identified 45.5 days (median, IQR = 41.5–50.5) after the estimated date of infection, at which time plasma samples were obtained from both the transmitting partner (donor) and the seroconvertor (recipient). The vast majority (95%) of HIV-1 sequences derived from ZEHRP transmission pairs belonged to HIV-1 subtype C with subtypes A, D, G, and J being detected only occasionally [60]. All of the transmission pairs utilized in this study are infected with subtype C HIV-1.

### Viral loads and CD4+ count measurements

Early set point VL for newly infected individuals was defined as the earliest stable nadir VL value measured between 3 and 9 months post infection and which did not show a significant increase in value within a 3–4 month window. HIV plasma VL was determined at the Emory Center for AIDS Research Virology Core Laboratory using the Amplicor HIV-1 Monitor Test (version 1.5; Roche). CD4+ T cell counts were based on T-cell immunophenotyping, with assays done using the FACScount System (Beckman Coulter Ltd., London, United Kingdom) in collaboration with the International AIDS Vaccine Initiative.

### HLA-class I genotyping

Genomic DNA was extracted from whole blood or buffy coats (QIAamp blood kit; Qiagen). HLA class I genotyping relied on a combination of PCR-based techniques, involving sequence-specific primers (Invitrogen) and sequence-specific oligonucleotide probes (Innogenetics), as described previously [10]. Ambiguities were resolved by direct sequencing of three exons in each gene, using kits (Abbott Molecular, Inc.) designed for capillary electrophoresis and the ABI 3130*xl* DNA Analyzer (Applied Biosystems).

### Amplification and sequencing of *gag* from donors and linked recipients

Viral RNA was extracted from 140 µL plasma samples using the Qiagen viral RNA extraction kit (Qiagen). *Gag-pol* population sequences were generated using nested gene specific primers. Combined RT-PCR and first round synthesis was performed using SuperScript III Platinum One Step RT-PCR (Invitrogen) and 5 µL viral RNA template. RT-PCR and first round primers include GOF (forward) 5′ ATTTGACTAGCGGAGGCTAGAA 3′ and VifOR (RT-PCR and reverse) 5′ TTCTACGGAGACTCCATGACCC 3′. Second round PCR was performed using Expand High Fidelity Enzyme (Roche) and 1 µL of the first round PCR product. Nested second round primers include GIF (forward) 5′ TTTGACTAGCGGAGGCTAGAAGGA 3′ and VifIR (reverse) 5′ TCCTCTAATGGGATGTGTACTTCTGAAC 3′. Three positive amplicons per individual were pooled and purified via the Qiagen PCR purification kit (Qiagen). Purified products were sequenced by the University of Alabama at Birmingham DNA Sequencing Core. Sequence chromatograms were analyzed using Sequencher 5.0 (Gene Codes Corp.), and degenerate bases were denoted using the International Union of Pure and Applied Chemistry codes when minor peaks exceeded at least thirty percent of the major peak height.

The percent similarity between donor and recipient population *gag* sequences was determined by building a neighbor-joining tree using Geneious v5.5.7 (Biomatters Ltd.). The percent similarity between nucleotide and amino acid alignment was determined based on the output matrix from these neighbor-joining trees. In calculating the percent similarity between amino acid sequences, degenerate bases that resulted in non-synonymous changes and, thus, a mixture of amino acid residues, were translated as an “X”. When one of the amino acids comprising a mixture in the donor was found in the recipient Gag sequence, this was counted as a mismatch, making the average percent similarity reported between donor and recipient *gag* sequences a maximal estimate of percent mismatch.

### Generation of Gag-MJ4 chimeras

Viral RNA was extracted from linked recipients at the day of seroconversion time point using the Qiagen viral RNA extraction kit (Qiagen). First round PCR products were generated as was previously described for the *gag* sequencing of all 149 transmission pairs [50]. Second round PCR was performed to generate *gag* amplicons for Gag-MJ4 chimera generation using Phusion Hot Start II polymerase (Fisher) and 1 µL of the first round PCR product. Nested second round primers include GagInnerF1 (forward) 5′ AGGCTAGAAGGAGAGAGATG 3′ and BclIDegRev2 (reverse) 5′ AGTATTTGATCATAYTGYYTYACTTTR 3′, which generate a *gag* amplicon starting from the initiation codon of *gag* and extending 142 nucleotides after the *gag* stop codon and into *pro*. The 5′ portion of the MJ4 long terminal repeat (LTR) was amplified using Phusion Hot Start II polymerase and the MJ4For1b (forward) 5′ CGAAATCGGCAAAATCCC 3′ and MJ4Rev (reverse) 5′ CCCATCTCTCTCCTTCTAGC 3′ primer set. In order to make the proper insert for cloning, the patient-specific *gag* and MJ4-LTR sequences were joined using a splice-overlap extension PCR utilizing the MJ4For1b (forward) and BclIRev (reverse) 5′ TCTATAAGTATTTGATCATACTGTCTT 3′ primer set. Joined splice-overlap-extension PCR products were gel purified using the Qiagen gel extraction kit (Qiagen). Purified Gag-LTR inserts and wild-type MJ4 vector (NIH AIDS Research and Reference Reagent Program, [56]) were digested with NgoMIV and BclI restriction enzymes (NEB) and ligated overnight at 4°C with T4 DNA ligase (Roche) at a 3∶1 insert to vector ratio. JM109 competent cells were transformed with ligation products, plated onto LB/agar plates supplemented with 100 µg/ml ampicillin and grown at 30°C. Gag-MJ4 chimeric DNA was isolated from cultures using the Qiagen miniprep kit (Qiagen). Gag-MJ4 chimeras were sequenced to confirm *gag* insert fidelity as compared to previously amplified population sequences. Two identical independent clones per linked recipient were chosen for replication assays in order to ensure backbone fidelity during the cloning process.

### Generation and titration of viral stocks

Viral stocks were generated by transfecting 1.5 µg purified proviral plasmid DNA into 293T cells (American Type Culture Collection) using the Fugene HD transfection reagent (Roche) according the manufacturer's protocol. Viral stocks were collected 72 hrs post transfection, clarified by low-speed centrifugation, and frozen at −80°C. The titer of each viral stock was determined by infecting TZM-bl cells (NIH AIDS Research and Reference Reagent Program) with 5-fold serial dilutions of virus in a manner previously described [36], [61].

Both 293T and TZM-bl cell lines were maintained in complete Dulbecco's modified Eagle's medium (DMEM, Gibco) supplemented with 10% fetal bovine serum (HyClone Laboratories), 2 mM L-glutamine, and 100 U/ml penicillin G sodium, and 100 µg/ml streptomycin sulfate (Gibco) at 37°C and 5% CO_2_.

### *In vitro* replication capacity (RC) assay

In order to assess the RC of Gag-MJ4 chimeras, 5×10^5^ GXR25 cells [62] were infected at an MOI of 0.05, and 100 µl of viral supernatants were collected at 2 day intervals. Briefly, GXR25 cells and virus were incubated with 5 µg/ml polybrene at 37°C for 3 hours, washed 5 times with complete Roswell Park Memorial Institute (RPMI) medium (Gibco) and plated into 24-well plates. Cells were split 1∶2 to maintain confluency, replaced with an equal volume of fresh media, and viral supernatants were taken at days 2, 4, 6, and 8 as previously described [36], [61]. Virion production was quantified using a ^33^P-labeled reverse transcriptase assay. Based on values obtained for days 2–8, the optimal window for logarithmic growth for all viruses was determined to be between days 2 and 6, as by day 8 many high replicating viruses had exhausted target cells causing a flattening or decline of the replication curve. Therefore, log_10_-transformed slopes were calculated based on days 2, 4, and 6 for all viruses. Replication scores were generated by dividing the log_10_-transformed slope of the replication curve for each Gag-MJ4 chimera by the log_10_-transformed slope of wild-type MJ4. Two independent Gag-MJ4 chimera clones per acutely infected linked recipient were run in duplicate to confirm cloning fidelity. After both independent clones were confirmed to have identical replicative capacities, one clone was subsequently run in triplicate in two independent experiments in order to generate consistent replicative capacity scores. GXR25 cells were maintained in complete RPMI medium supplemented with 10% fetal bovine serum (HyClone Laboratories), 100 U/ml penicillin G sodium, 100 µg/ml streptomycin sulfate (Gibco), and 10 mM HEPES buffer at 37°C and 5% CO_2_.

### Quantification of HIV-1 reverse transcriptase

Aliquots of culture supernatants from infected cells were added to an RT-PCR master mix [63] and incubated at 37°C for 2 hours; then the RT-PCR product was blotted onto DE-81 paper, and allowed to dry. Blots were washed 5 times with 1× SSC (0.15 M NaCl, 0.015 M sodium citrate, pH 7.0) and 3 times with 90% ethanol, allowed to dry, and exposed to a phosphoscreen (Perkin Elmer) overnight. Counts were read using a Cyclone PhosphorImager [36], [61].

### HIV-1 Gag polymorphism scores

HLA-associated polymorphisms were defined as any non-consensus polymorphism that occurred at an amino acid position having known escape mutations adapted to specific HLA-class I alleles as defined by a list of associations generated in a manner similar to that described previously [44], [64] from 1899 subtype C *gag* sequences from Zambia and South Africa (Carlson, *et al.*, manuscript in preparation). Polymorphisms that increased or decreased replicative capacity were defined based on amino acid associations with RC derived from an exploratory pair-wise analysis detailed in the experimental results. To generate a summed polymorphism score, the number of HLA-associated fitness-decreasing polymorphisms was subtracted from the number of HLA-associated fitness-increasing polymorphisms.

### Statistical analysis

The relationships between RC and set point VL, donor VL, and average CD4+ counts; RC and the number and quality of HLA-associated polymorphisms; and VL and the number and quality of HLA-associated polymorphisms were analyzed using the Spearman rank correlation. Linear regression analyses were utilized to generate trend lines to facilitate visualization of correlation graphs. Mann-Whitney tests were used to compare the differences in RC between rare and more common polymorphisms. Mann-Whitney tests were used to analyze the difference in median set point VLs between different RC groups (RC<1, RC = 1–2, RC>2). All Spearman correlations, Mann-Whitney tests, and linear regression analyses were performed using Prism GraphPad v5.0 (GraphPad Software, Inc.).

The Mann-Whitney U test was used to identify statistically significant differences in RC between two groups (e.g. sequences with or without a given HIV polymorphism). Multiple tests were addressed using q-values [65], which estimates the expected proportion of significant tests that are false positives. To limit the number of tests, we considered only groups containing at least 3 individuals.

A subset of volunteers with longitudinal CD4+ T cell counts (n = 63) was analyzed to characterize the relationship between RC and T cell decline. Survival analysis was used to estimate the association between replicative capacity (RC) and the drop of CD4+ cell count. The endpoint is defined as the time before CD4+ T cell counts drop below a threshold, e.g. 300 or 350 cells/mm^3^. The set point VL is another factor being considered. Kaplan Meier curve and log-rank test were used to compare the survival between the groups with RC<1 and RC>2. Cox proportional hazard regression was used to assess risk associated with high RC with or without adjustment for the confounding factor of set point VL. The sample size (n = 66) was inadequate for a more complete analysis with additional covariates.

## Results

### Selection and characterization of transmitted *gag* sequences

Studying heterosexual transmission of HIV-1 within the context of discordant couples allows for full characterization of viruses from both donor and recipient. From a total of 294 epidemiologically linked transmission pairs identified from 1998–2010 in the Zambia Emory HIV Research Project (ZEHRP) discordant couple cohort [66], we selected 149 individuals that had been enrolled for at least 1 year and had at least 9 months of follow up post serconversion. The median time post estimated date of infection (EDI) for all 149 individuals was 45 days. Thus, this represents a unique early infection linked transmission pair cohort.

The median log_10_ VL for donors near the time of transmission and the median log_10_ set point VL for linked recipients was 5.02 (IQR = 4.51–5.45) and 4.39 (IQR = 3.91–4.99) respectively for all participants included in this study. Figure S1 depicts the phylogenetic clustering of population sequences for the *gag* gene of the 149 epidemiologically linked transmission pairs and highlights the high degree of sequence similarity between donor and recipient viruses.

The majority of population *gag* sequences isolated from acute/early time points of linked recipients were homogeneous and in most cases were identical to the donor population *gag* sequence isolated near the EDI. Overall, donor and linked recipient sequences differed in amino acid composition by only 2.7%. Mutations were counted when a mixture of nucleotides (amino acids) in the donor population sequence resolved to a single residue in the recipient and thus represent maximal values. Of the 149 pairs only 37 recipient sequences had evidence of potential early escape and a majority of these individuals (28/37) had only a single amino acid change. Therefore, we can conclude that the majority of the sequence polymorphisms present at the seroconversion time point are derived from the chronically infected donor. Additional characteristics of the cohort including set point VL and CD4+ counts are listed in Table 1.

**Table 1:** Cohort statistics generated from the 149 transmission pairs selected from the ZEHRP cohort.

Parameters	Linked Seroconverter (SC)
Number of individuals	149
Number of Females (%)	77 (52%)
Age of SC at time of seroconversion	28 (24–35)^a^
Estimated days post infection	45.5 (41.5–50.5)^a^
Log_10_ Set-point VL of SC	4.39 (3.91–4.99)^a^
Log_10_ VL of Donor Partner^b^	5.02 (4.51–5.45)^a^
CD4 cell count 1 yr post SC^c^	383 (308–503)^a^

a Median (Inter-Quartile Range).

b Measured at or within one month of seroconversion of LR.

c LR for whom we have longitudinal CD4+ T cell counts (n = 63).

### Construction of Gag-MJ4 chimeric viruses

Previous studies investigating the role of Gag viral fitness have employed a recombination approach in which sequences are PCR amplified as a bulk population, allowed to recombine into a *gag-deleted* NL4-3, and resulting viruses propagated in permissive cells [43], [47], [51], [53]–[55]. This method has three distinct disadvantages: there is no control over the sites of recombination, it requires the outgrowth of virus which may select for the most fit virus in the population and could also select for sequence changes, and the introduction of subtype C sequences into a lab adapted subtype B proviral backbone may introduce biases due to the interaction of subtype B proteins with subtype C Gag. In order to avoid these limitations and because we are studying individuals recently infected with HIV, where the population is generally homogeneous, we have employed a direct cloning method that allows for the introduction of the entire *gag* gene into a replication competent, CCR5 tropic, clade C provirus, MJ4 [56].

A splice-overlap-extension PCR was employed to fuse the MJ4-LTR-U5 sequence with the transmitted *gag* sequence. This ensures that the cis-acting sequences upstream of Gag, which may influence expression levels, are constant throughout all constructs. The resulting chimeras include the entire transmitted *gag* sequence from the initiation codon to the end of Gag and extend into conserved region of protease by 47 amino acids. For each newly infected individual, at least two independent Gag-MJ4 chimeras were sequence confirmed and assayed for replicative capacity (RC). An analysis of variation of RC between the two independent clones derived from each newly infected linked recipient was 8.5%. Testing two independent clones, therefore, ensures that the observed RC is not due to the confounding effect of backbone mutations that might have arisen during the cloning process and provides an estimate of experimentally induced variation.

Overall, a low amount of heterogeneity was detected in *gag* population sequences isolated from linked recipients, with only 28% having one or more mixed bases resulting in amino acid changes in the sequences from which the Gag-MJ4 chimeras were generated. When this was the case, the *gag* clone with the sequence closest to the donor *gag* sequence was chosen in order to avoid sampling *gags* containing *de novo* escape or reversion. In some cases in which multiple variants appeared to be transmitted, several *gag* variants were assayed for RC as described in the materials and methods section. In each case, these minor variants were found to have similar or identical RC values (data not shown).

### Introduction of *gag* sequences from newly infected individuals into MJ4 drastically alters replicative capacity

**Figure 1:**
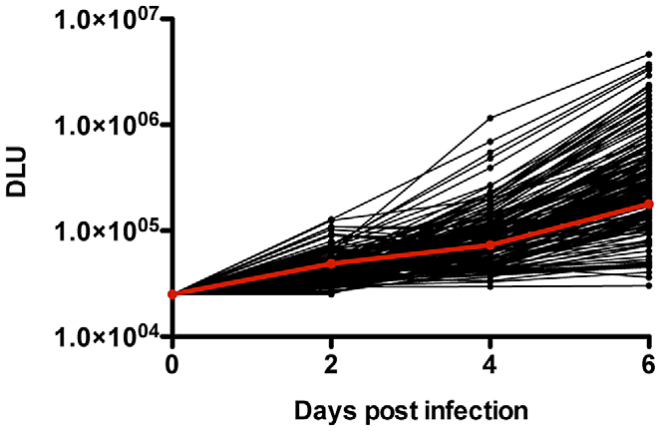
Insertion of the *gag* gene from newly infected individuals dramatically alters the replicative capacity of MJ4. In order to generate replication curves, 5×10^5^ GXR25 cells were infected with Gag-MJ4 chimeras at an MOI of 0.05. Viral supernatants (100 µL) were collected on days 2, 4, and 6, and the amount of virus in supernatants was quantified using a radiolabeled reverse transcriptase assay. Replicative capacity scores were generated by dividing the log_10_-transformed slope of replication of each Gag-MJ4 chimera by the log_10_-transformed slope of MJ4. It is clear that the insertion of the *gag* gene alone can dramatically alter the *in vitro* RC, generating viruses with up to 1000-fold different replication curves. DLU, digital light units.

In initial replication assays performed in order to test assay precision, wild-type MJ4 exhibited an intra-assay variability of 10.4% and an inter-assay variability of 8.7%. Figure 1 shows the results of a typical experiment for all 149 Gag-MJ4 chimeras, with wild-type MJ4 depicted in red. The normalized RC values of the chimeras ranged from less than 0.01 to greater than 3.5. Some viruses replicated more than 100 times more efficiently than MJ4, demonstrating that substitution of Gag can have a profound impact on the ability of the virus to replicate in cells.

### Correlation between the replicative capacities conferred by transmitted *gag* sequences and viral loads in newly infected individuals and their transmitting partners

**Figure 2:**
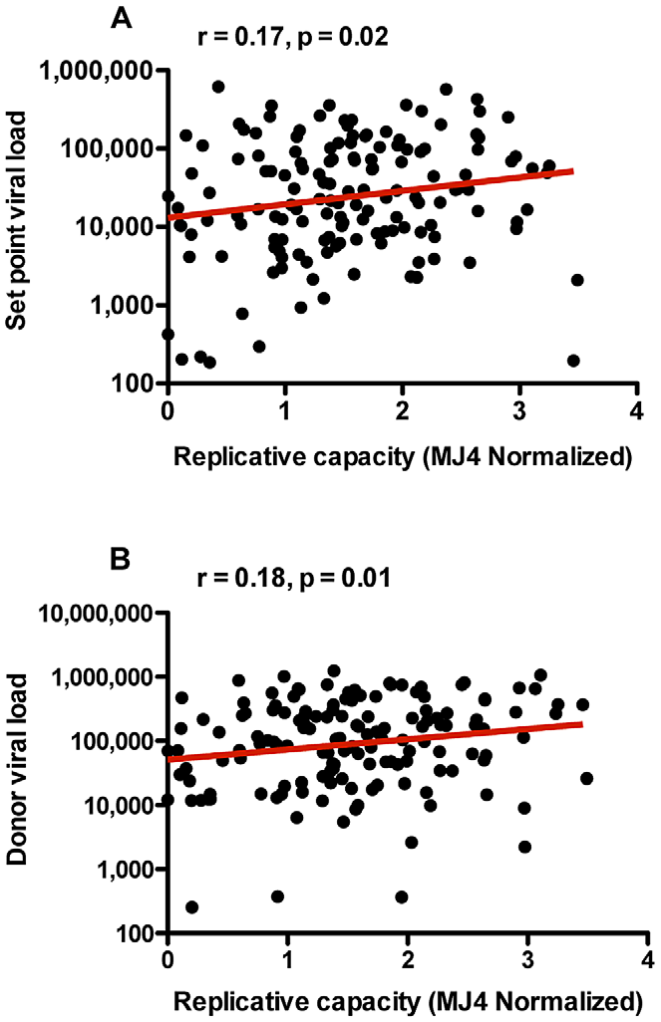
Replicative capacity is correlated to viral load in recipients and donors. (A) The RC of Gag-MJ4 chimeras generated from *gag* sequences isolated from epidemiologically linked recipients at acute time points correlates to early set point VL in the same acutely infected recipients (Spearman correlation, r = 0.17, p = 0.02). Replicative capacity scores were generated by normalizing the log_10_-transformed slopes of replication curves from days 2 through 6 for each Gag-MJ4 chimeric virus to the log_10_-transformed slope of wild-type MJ4. (B) The RC of Gag-MJ4 chimeric viruses also correlates to the VL near the estimated date of infection in chronically infected donors (Spearman correlation, r = 0.18, p = 0.01). Trend lines were generated using linear regression analysis, and are shown in order to facilitate visualization of correlations.

An examination of the RC of transmitted viruses allows us to determine the role of viral replication in defining set point VL in acutely infected individuals before significant viral adaptation to immune pressure of the host has taken place, which might confound the relationship of RC to VL. We observed a statistically significant positive correlation between the replicative capacities of Gag-MJ4 chimeras and set point VLs in newly infected individuals (Figure 2A; Spearman correlation r = 0.17, p = 0.02), a correlation that persists when conditioning on the presence of B*57 in, and the sex of, the linked recipient (p = 0.009). This finding indicates that the RC conferred by the transmitted Gag sequence clearly plays a role in defining early set point VL of newly infected Zambian linked recipients.

In several cohorts VL in the transmitting partner and that in the linked seroconvertor have been shown to be correlated [11]–[13]. In order to more fully explore the possible contribution of RC in explaining this phenomenon, we compared VLs of the transmitting partner at the time of transmission to the RC defined by the transmitted Gag sequence. Despite both a higher maximum and wider range of VLs within transmitting partners, we observed a statistically significant positive correlation between RC and the set point VL of the donors, similar to that of their newly infected partners (Figure 2B; Spearman correlation r = 0.18, p = 0.01). This supports the concept that RC, defined by Gag, is a viral characteristic contributing to the positive correlation between donor and recipient VLs that has been previously reported [11]–[13].

### Several amino acids in Gag significantly correlate to changes in replicative capacity

Uncovering sites of vulnerability in HIV-1 is a high priority for the informed design of an effective HIV vaccine [21]. Therefore, we examined all 149 Gag sequences and their RC using an exploratory pairwise analysis described previously [43], [54], to uncover residues that significantly affect the virus' ability to replicate *in vitro*. We found 49 residues at 31 unique positions that had a statistically significant effect on RC at p<0.05 (q<0.51) and 4 residues at 3 unique positions that were significant at p<0.002 (q<0.2) (Table S1). In what follows, we will use q<0.2 as the significance threshold when individual sites of significance are considered, and q<0.51 (p<0.05) as the significance threshold when we are testing broad trends, in which we are willing to increase our expected false positive rate as a tradeoff to substantially reduce our expected false negative rate.

**Figure 3:**
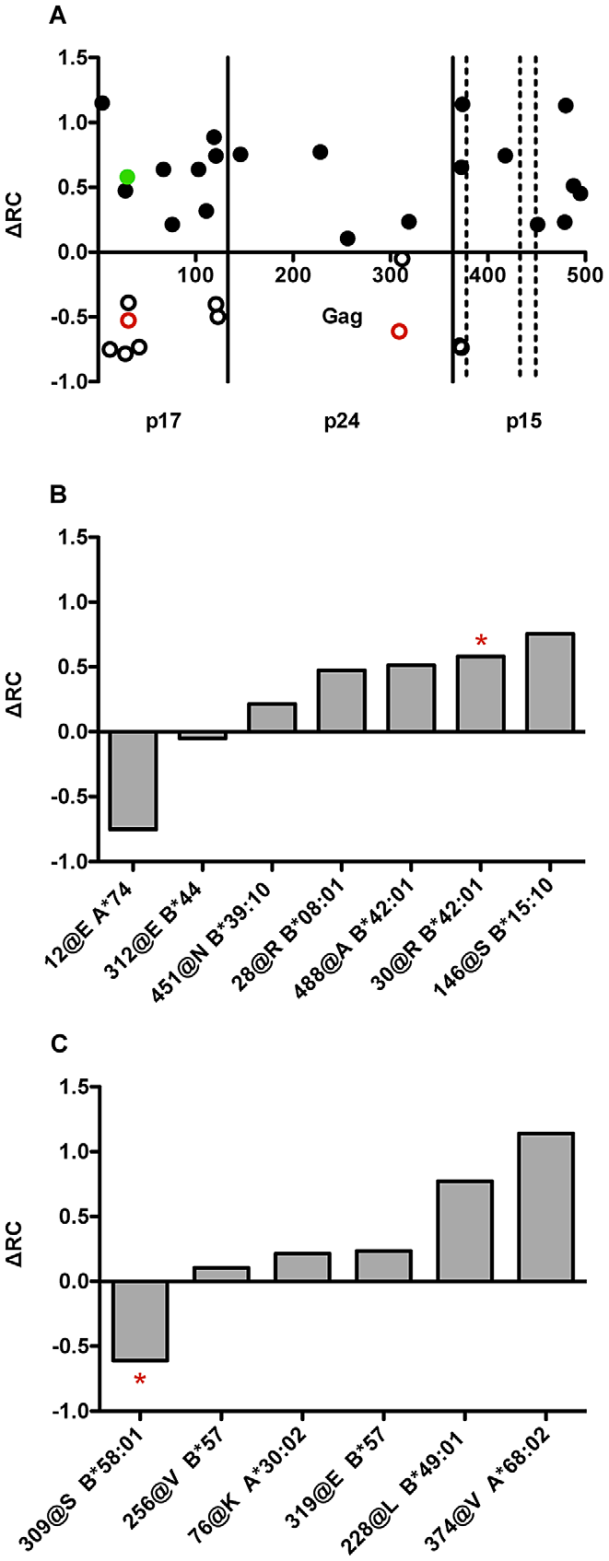
Identification of polymorphisms in Gag that significantly affect RC, several of which can be linked to HLA-class I alleles. (A) In a pair-wise analysis of all amino acids represented in the 149 *gag* sequences tested, we observed 49 amino acids at 31 unique positions that were statistically associated (p<0.05) with changes in RC (open circles RC decreasing, filled circles RC increasing), 3 of which were significant at (q<0.2) when adjusted for multiple comparisons (red for RC reducing and green for RC increasing). The x-axis shows the polymorphism position in the primary Gag sequence (HXB2 numbering), and the y-axis depicts the impact of the polymorphism on RC relative to the median RC of all viruses (∼1.5). (B) In a separate study analyzing 1899 subtype C gag sequences from Zambia and South Africa, 199 residues were linked to HLA-I alleles (q<0.2, Carlson, Schaefer *et al.*, manuscript in preparation). From this, a total of 7 polymorphisms associated with changes in RC (p<0.05) were found to be adapted to specific HLA-I alleles, adapted (amino acid is present when HLA-I allele is also present.) (* denotes q<0.2). (C) Six polymorphisms associated with changes in RC (p<0.05) were found to be non-adapted (amino acid is present only when HLA-I allele is absent) to specific HLA-I alleles (* denotes q<0.2).

The locations of all statistically significant polymorphisms (p<0.05), along with their effects on RC as compared to the median RC of all viruses, are plotted linearly on a graphical representation of the Gag protein (Figure 3A.). Residues that dramatically modulate RC were enriched in p17 and p2 (Fisher's exact test, p<0.0001). In addition, roughly two-thirds of the non-consensus residues with p<0.05 increase fitness relative to the median RC for the entire population.

An expanded data set of 1899 subtype C *gag* sequences from Zambia and South Africa (Carlson, Schaefer et. al., manuscript in preparation) was utilized to identify residues that affected RC and were also HLA-associated. Within this data set, HLA-associated polymorphisms are classified as being either adapted or non-adapted. An adapted residue is one that is escaped relative to the HLA-allele in question. In contrast, a residue that is non-adapted is the susceptible form and may render the virus vulnerable to immunological targeting by the HLA-allele in question. This new dataset has identified a total of 199 HLA-linked polymorphisms (q<0.2, p<0.0007) vs. 59 associations utilized previously from a smaller subset of *gag* sequences [27], [50]. Within the 49 residues associated with changes in RC, 7 polymorphisms were found to be adapted to specific HLA class I alleles, clearly demonstrating the impact of the cellular immune response in affecting viral fitness (Figure 3B, * denotes q<0.2). Six polymorphisms were found to be non-adapted to specific HLA class I alleles (Figure 3C, * denotes q<0.2). Since these are non-consensus polymorphisms, it is possible that consensus at these residues is escaped relative to these HLAs, potentially explaining why an adapted consensus residue at this position is the less fit variant. Indeed, 5 consensus residues (62K, 451S, 488S, 85L, and 309A) with p<0.05 were found to be adapted to HLA-I alleles, demonstrating that the cellular immune response can drive selection for consensus residues.

### Rare polymorphisms have the greatest effect on replicative capacity

**Figure 4:**
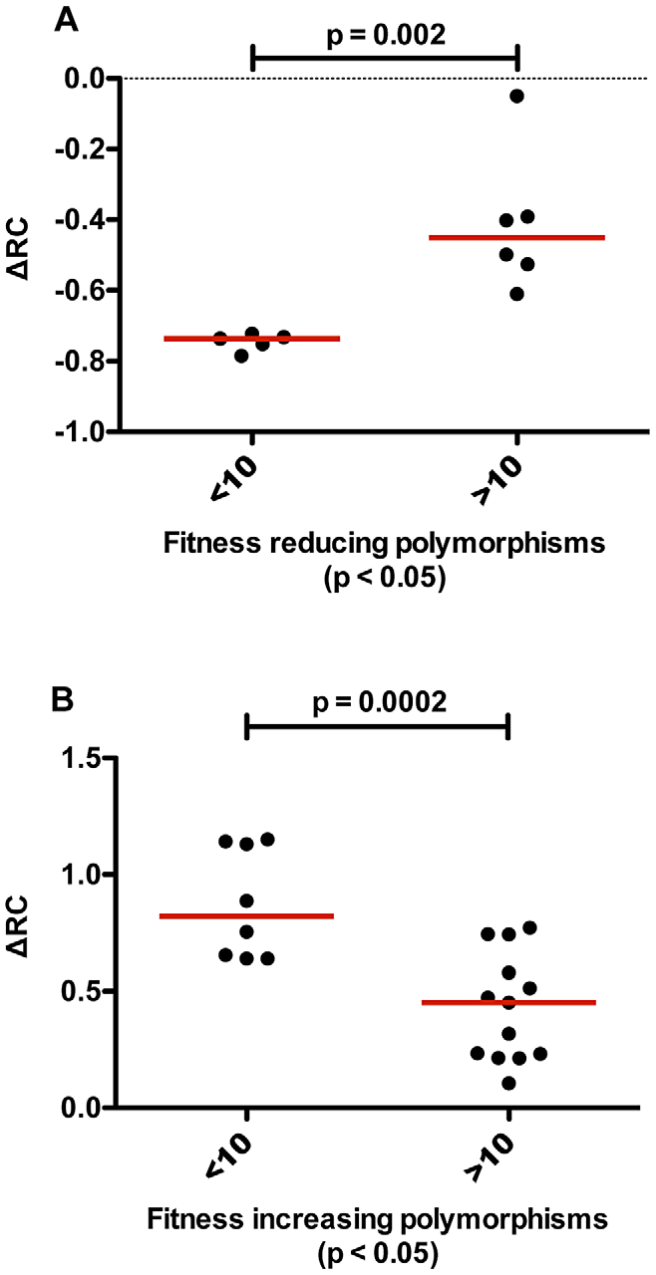
Rare polymorphisms have a significantly greater impact on RC. (A) In an exploratory analysis, rare fitness reducing polymorphisms, occurring in less than 10 out of 149 of the sequences tested, were found to decrease fitness to a significantly greater degree than more common fitness decreasing mutations (Mann Whitney U test, p = 0.002). The y-axis depicts the impact of the polymorphism on RC relative to the median RC of all viruses (∼1.5) (B) Rare fitness increasing polymorphisms, occurring in less than 10 out of 149 of the sequences tested, were found to increase fitness to a significantly greater degree than more common ones (Mann Whitney U test, p = 0.0002).

During our analysis of amino acid polymorphisms linked to changes in RC (p<0.05), we observed a negative correlation between the frequency of polymorphisms and the magnitude of their effect on RC (Spearman correlation, r = −0.89, p<0.0001). Indeed, rare polymorphisms, those occurring in less than 10 of the 149 individuals studied, had significantly greater impact (both negative and positive) on RC than polymorphisms that occurred more frequently (Figure 4A and 4B). This finding is especially intriguing in the case of rare deleterious mutations, as these residues may highlight epitopes at which HIV escapes or compensates for fitness defects with great difficulty, similar to those described for elite controllers [52], and may, therefore, be attractive targets for a cellular-based vaccine.

### The cumulative and qualitative effect of HLA associated polymorphisms in Gag on replicative capacity

**Figure 5:**
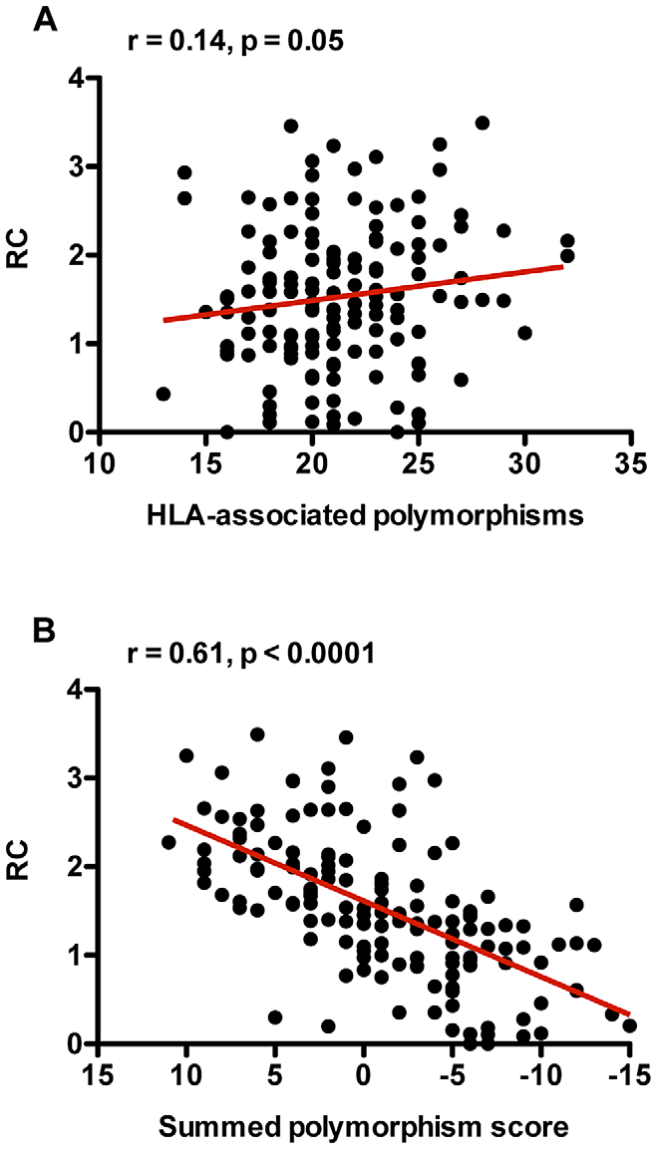
The balance of fitness increasing and decreasing HLA-associated polymorphisms strongly correlates with RC. (A) The total number of HLA-associated polymorphisms positively correlates with RC. HLA-associated polymorphisms were defined as non-consensus residues at any amino acid position known to have polymorphisms statistically linked to HLA-I alleles. (B) RC of Gag-MJ4 chimeras is highly correlated to the summed polymorphism score in Gag. Each amino acid polymorphism was given a score of 1 (fitness increasing) or −1 (fitness decreasing). Summed polymorphism scores were generated by summing these scores for each Gag protein. Trend lines were generated using linear regression analysis, and are shown in order to facilitate visualization of correlations.

In order to investigate whether there is a cumulative effect of viral escape from cellular immune pressure in Gag on RC, the expanded dataset of HLA-associated polymorphisms generated from an analysis of 1899 *gag* sequences from Zambia and South Africa (Carlson, Schaefer *et al.*, manuscript in preparation), described above, was employed. The number of non-consensus polymorphisms located at HLA-associated positions was determined for each MJ4 chimera and then correlated with the RC defined by those Gag sequences. Surprisingly, we found a positive association between the number of HLA-associated polymorphisms and RC (Fig. 5A; r = 0.14, p = 0.05). Although counterintuitive, this is consistent with the fact that not all HLA-associated polymorphisms within a particular Gag sequence will necessarily reduce fitness. We have shown in the previous sections that several non-adapted (or “susceptible” to HLA pressure) HLA-associated polymorphisms increase fitness relative to the median of all sequences. Indeed, we observe a highly statistically significant positive correlation between the number of non-adapted HLA-associated polymorphisms and RC (Spearman correlation, r = 0.23, p = 0.003; data not shown). Thus, the inclusion of both adapted (or escaped with respect to specific HLA alleles) and non-adapted polymorphisms within this expanded HLA-associated dataset may explain the observed positive association between numbers of HLA-associated polymorphisms and RC. Therefore, we hypothesize that it is the balance and interaction of both fitness increasing and fitness decreasing polymorphisms within a particular sequence that ultimately determines the RC of the virus.

In order to more accurately determine how the number and quality of HLA-associated polymorphisms affects RC and to correct for the opposing influence of both increasing and decreasing polymorphisms within a particular sequence, a summed polymorphism score was calculated by assigning fitness increasing polymorphisms a score of +1, fitness decreasing polymorphisms a score of −1, and neutral polymorphisms a score of 0. HLA-associated polymorphisms were defined as being positive, negative, or neutral based on the previously described univariate analysis that correlated specific residues within our 149 sequences with changes in RC. In this modified analysis, we observed a highly statistically significant correlation between the summed polymorphism score and RC (Figure 5B: Spearman rank correlation; r = 0.6, p = <0.0001), confirming that the sequence features are approximately independent of each other and suggesting that the offsetting influence of fitness decreasing and increasing polymorphisms is a strong contributor to RC. This finding may explain the observation that, in general, the most-fit viruses are less like the consensus subtype C Gag sequence, consistent with a majority of polymorphisms increasing fitness (Figure S2, [55]).

### The cumulative and qualitative effect of HLA-associated polymorphisms in Gag on set point viral load

**Figure 6:**
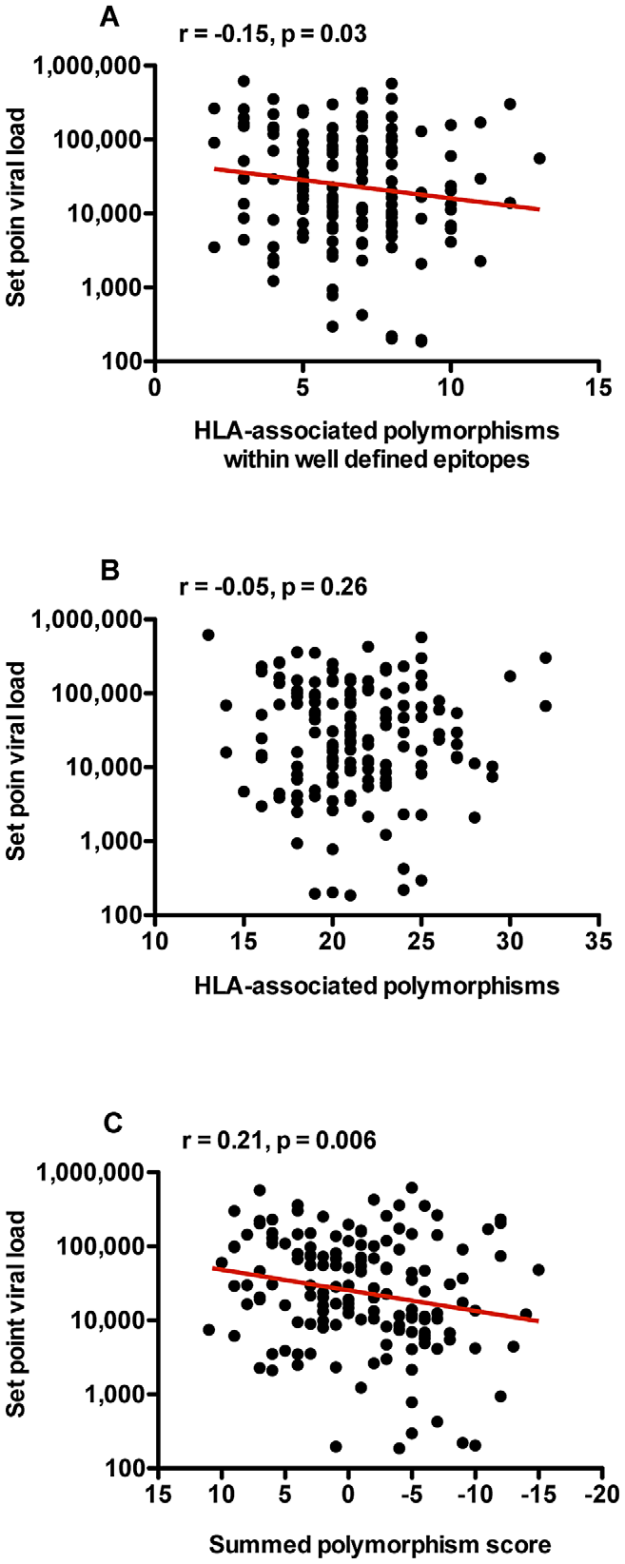
The balance of HLA-associated fitness increasing and decreasing mutations strongly correlates with set point viral load in newly infected individuals. (A) The number of HLA-associated amino acid polymorphisms within well-defined epitopes for each Gag protein negatively correlates with set point VL. (B) The total number of HLA-associated polymorphisms (including those outside well-defined CTL epitopes) in Gag does not correlate to set point VL in newly infected individuals. (C) When the quality of HLA-associated polymorphisms is considered, a strong correlation between the summed polymorphism score (as defined in Figure 5) and set point VL is observed. Trend lines were generated using linear regression analysis, and are shown in order to facilitate visualization of correlations.

In a previous report using 88 Zambian linked seroconverters, we reported that increasing numbers of transmitted HLA-B associated polymorphisms within or adjacent to well defined epitopes were associated with lower set point VLs [50]. When we expand this analysis to include all 149 Zambian linked recipients and use the same dataset of HLA-linked polymorphisms used by Goepfert *et al.* [50] we observe the same correlation (r = −0.15, p = 0.03, Figure 6A). However, when we use the expanded HLA-associated data set (199 associations) to define HLA-associated polymorphisms, we no longer observe a statistically significant negative association between the number of transmitted HLA-associated polymorphisms in Gag and set point VL (Figure 6B).

We therefore hypothesized that, as with RC, this correlation between the total number of transmitted HLA-associated polymorphisms in Gag and set point VL in newly infected individuals may be confounded by not taking into account whether polymorphisms increase or decrease fitness. Indeed, using the summed polymorphism score, we observe a highly significant correlation between the summed score of HLA-associated polymorphisms and set point VL (Figure 6C: r = 0.21, p = 0.006). This demonstrates that it is not merely the quantity of HLA-associated polymorphisms present in the transmitted Gag sequence that ultimately defines set point VL, but it is the influence of both fitness increasing and decreasing polymorphisms that contributes to RC and in turn set point VL in newly infected individuals.

### Transmission of viruses with low replicative capacities provides recipients with a longer-term clinical benefit

**Figure 7:**
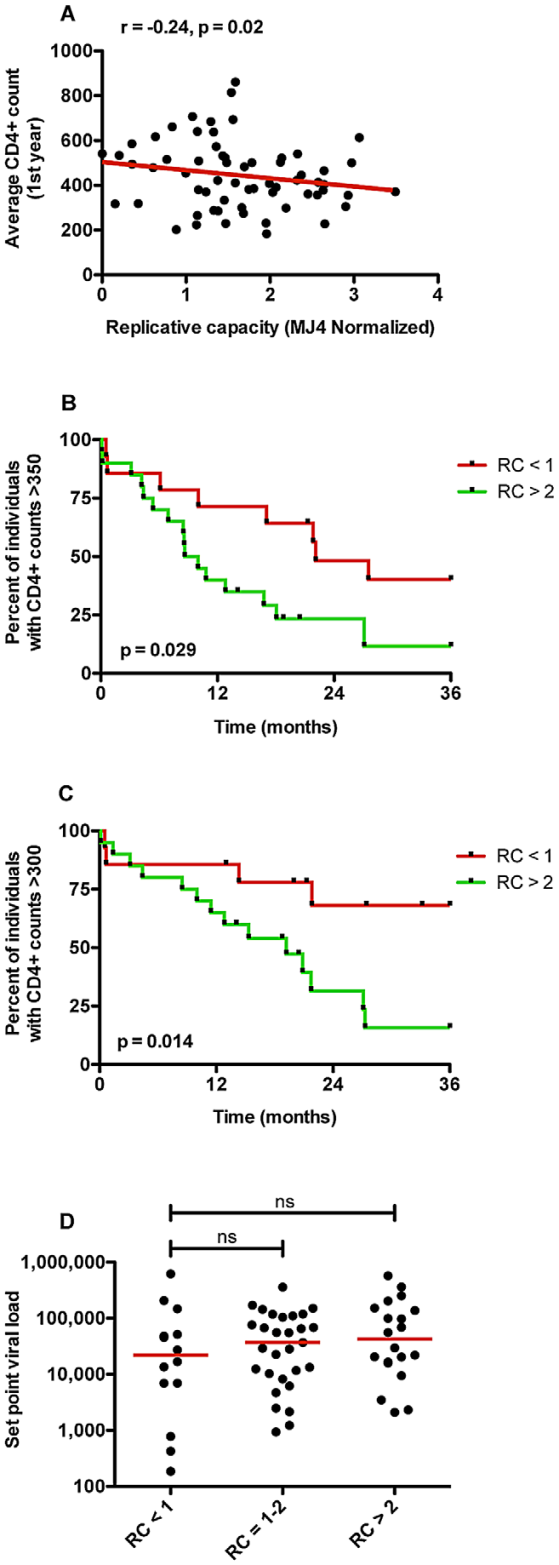
RC affects the rate of CD4 decline in a manner that may be independent of viral load. (A) In a subset of individuals for which longitudinal CD4+ counts were available for at least 1 year post infection (n = 63), RC of Gag-MJ4 chimeras negatively correlated with the average CD4+ counts of linked recipients within the first year (Spearman rank correlation, r = −0.24, p = 0.02). (B) Kaplan-Meier plots in which the endpoint was defined as the first CD4+ count below 350. Interval cut-off for endpoint was set to 36 months. The difference in median time to endpoint between those receiving viruses with RC<1 (n = 14) and RC>2 (n = 20) was 384 days (Log-rank test, p = 0.029). (C) Kaplan-Meier plots in which the endpoint was defined as the first CD4+ count below 300. Interval cut-off for endpoint was set to 36 months. The difference in median time to endpoint between those receiving viruses with RC<1 (n = 14) and RC>2 (n = 20) was >800 days (Log-rank test, p = 0.014). (D) The difference in median VL between RC groups RC<1, RC 1–2, and RC >2 were not significantly different (Mann Whitney U test), consistent with the effect of RC on CD4 decline being independent of VL.

Though set point VL has been shown to be a relevant marker for disease progression [67], [68], CD4+ T cell counts are traditionally used to define those individuals that have progressed to AIDS and are at a higher risk for opportunistic infections [69], [70]. Therefore, we analyzed a subset of individuals (n = 66) for whom longitudinal CD4+ T cells counts for at least one-year post-infection are available. We observed a statistically significant correlation between the average CD4+ T cell counts and the replicative capacities of Gag-MJ4 chimeras (Figure 7A, Spearman correlation, r = −0.24, p = 0.02). This demonstrates that infection with attenuated viruses may impart some survival benefit to newly infected individuals, at least within the first year of infection.

Subsequently, we determined whether individuals infected with poorly replicating viruses exhibit differential pathogenesis over the first three years of infection or whether this early benefit is transient and quickly lost. To answer this question, we studied a subset of the linked recipients (n = 63) for whom CD4+ T cell counts were available at regular three month intervals for greater than one year post-infection.

In a Kaplan-Meier survival analysis, in which we defined the endpoint as having a CD4+ T cell count >350 cells/mm^3^ (WHO recommendation for initiation of anti-retroviral therapy [71]), we observed a statistically significant difference in the number of individuals that maintain CD4+ counts >350 cells/mm^3^ between those infected with viruses that replicate very poorly (RC<1) and those infected with highly replicating viruses (RC>2), within the first 3 years of infection (Figure 7B, Mantel-Cox test p = 0.029). This disparity in disease progression was even more pronounced when the endpoint was defined as having CD4+ T cell counts >300 cells/mm^3^, demonstrating a median difference of 896 days before falling below the CD4+ count cut off between individuals infected with low and high replicating viruses (Figure 7C, Mantel-Cox test p = 0.014). Using a Cox proportional hazard model, we demonstrate a significantly increased risk of CD4+ T cell counts falling below 350 (Hazard Ratio (HR) 2.36; p = 0.034) or 300 (HR 3.80; p = 0.021) over the first three years of infection for individuals whose Gag conferred an RC>2 vs. RC<1.

Interestingly, the benefit conferred by low replicating viruses could not be wholly explained by differences in set point VL within this smaller data set. Although there was a trend towards higher VLs between the two most disparate groups, with a 2.5 fold difference in median VLs (Figure 7D), we observed no statistically significant differences in median set point VLs between individuals infected with low (RC<1), medium (RC = 1–2), and highly (RC>2) replicating viruses. Further, in Cox proportional hazard models that take into account VL, the HR remained high (2.18 and 3.12 respectively) and p values continued to trend or remain borderline significant (p = 0.093 and 0.051) (Table 2), indicating that both VL and RC can independently affect CD4 loss. Moreover, the HR associated with log10 increases in set point VL alone was lower than that for RC alone (HR = 1.75 versus 2.62, and 2.09 versus 3.80; CD4 <350 and 300 respectively; Table 2). These results suggest that infection with a low replicating virus confers clinical benefit outside of the effect of RC on set point VL, and that the kinetics of viral replication early in infection can ultimately dictate long-term pathogenesis.

**Table 2:** Cox proportional hazard models demonstrate the independent effects of RC and VL on CD4 decline.

Risk of CD4 below 350 in first 3 years	p	Hazard Ratio^c^	lower .95^d^	upper .95^d^
RC>2^a^	0.034	2.62	1.077	6.374
Log10(SetPoint VL)^b^	0.04	1.75	1.014	3.004
RC>2+Log10(SetPointVL)	0.093	2.18	0.878	5.395

Risk of CD4 below 300 in first 3 years	p	Hazard Ratio^c^	lower .95^d^	upper .95^d^
RC>2^a^	0.021	3.80	1.226	11.801
Log10(SetPoint VL)^b^	0.023	2.09	1.105	3.94
RC>2+log10(SetPointVL)	0.051	3.12	0.995	9.751

a as compared to RC<1.

b Model in which log_10_ set point viral load in the linked recipient is taken into account.

c The ratio of hazard rates between the variables RC>2 and RC<1.

d The upper and lower bounds of confidence.

## Discussion

In this study of 149 linked Zambian heterosexual transmission pairs from the ZEHRP discordant couple cohort we have more fully characterized the role that HIV-1 viral characteristics, in particular the replicative capacity (RC) conferred by the transmitted *gag* sequence, plays in defining parameters of early HIV-1 pathogenesis. Identification and comparison of both donor and recipient *gag* sequences for all 149 transmission pairs revealed a high degree of similarity (97.6%) within each linked pair, allowing us to conclude that the majority of polymorphisms in Gag present at the seroconversion time point were transmitted from the chronically infected donor.

### Transmitted *gag* sequences from newly infected individuals engineered into MJ4 drastically alters replicative capacity

Since all of the transmission pairs in this study were infected with subtype C viruses, our approach of precisely cloning *gag* genes from acutely-infected recipients into a primary isolate (MJ4) provirus has many important advantages over previously employed methods. MJ4 is a CCR5 tropic infectious molecular clone derived from a subtype C clinical isolate from Botswana, providing greater homology to viruses circulating within the Zambian population than other previously used subtype B lab-adapted strains [33], [47], [54], [55]. Additionally, this cloning method for generating Gag-MJ4 chimeric viruses does not rely on recombination based technologies that require the outgrowth of viral quasispecies, which may select for the most fit virus, and in some cases, amino acid changes in the viral stocks that are not present in the individual from which they were derived [32], [47], [53]–[55]. The use of a common BclI restriction site located 137 nucleotides after the *gag* stop codon in MJ4 does result in a chimeric protease, however, this region is 96.5% conserved in this cohort and we did not observe a high prevalence of dead or inactive Gag-MJ4 chimeras.

The impact of engineering foreign *gag* sequences into MJ4 on virus replication was highly significant, with many of the chimeras exhibiting RC values greater than a hundred-fold higher than wild-type MJ4, which in this assay is one of the poorer replicators. This indicates that substitution of Gag can drastically alter the *in vitro* RC of the virus when all other viral components are constant. Multiple intra-molecular contacts as well as host protein interactions in Gag are necessary for effective intracellular Gag trafficking [72], [73], particle formation [74], budding [75], [76], maturation [77] and disassembly [78]. Therefore, immune mediated adaptation of this functionally constrained protein could have clear consequences for viral replication through disruption of these many interactions.

### Correlation between the replicative capacities conferred by transmitted *gag* sequences and viral loads in newly infected individuals and their transmitting partners

It has been well established that the set point VL in those recently infected with HIV-1 is correlated to disease progression and clinical outcome [67], [68]. Previous data from our group demonstrated that transmission of sequences with increasing numbers of CTL escape mutations in Gag resulted in lower set point VLs in newly infected individuals, a finding that suggested that transmitted HLA-associated polymorphisms in Gag might negatively affect viral replication [50]. We have confirmed this association in the current study after increasing the number of transmission pairs analyzed from 88 to 149. This result is consistent with studies by Brockman *et al.*, which have demonstrated a statistically significant link between the RC conferred by *gag-pro* genes in subtype B and C chronically infected individuals to VL [43], [47], [54], [55]. However, a statistically significant correlation between Gag RC derived from acutely infected individuals and set point VL has not previously been definitively reported in a subtype C cohort.

In contrast, in this large group of very early ZEHRP seroconvertors (with samples drawn a median of 45 days post-EDI) we observed a clear statistically significant correlation (p = 0.02) between the RC conferred by the transmitted *gag* sequence and the early set point VL in newly infected individuals. This result implies that RC plays a role in defining the overall level of virus replication during the first year of infection. Moreover, in multivariable analyses that take into account the early viral control imposed by the B*57 allele and by gender, the impact of RC on set point VL was found to be independent of these two host factors (p = 0.009). Other factors such as NK cells and restriction factors such as TRIM or APOBEC may potentially affect RC and VL, however little is currently known regarding these potential effects, and future efforts should evaluate the role of such factors. While we observed a statistically significant positive correlation between RC and set point VL, outliers in the data exist that do not fit the trend, and in some cases can be explained by the presence of protective HLA-alleles or by a large number of escape mutations present in the transmitted sequence that are relevant to the HLA background of the newly infected individual. Set point VL is clearly determined by a combination of both host factors, including HLA-alleles, and viral factors such as RC, and this may explain the differences in the absolute correlation for each individual.

The RC of Gag-MJ4 chimeras also correlated with VLs near the estimated date of infection in chronically infected donors, consistent with the previously reported observation that donor and recipient VLs are correlated within epidemiologically linked transmission pairs [11]–[13]. The data presented here would suggest that the RC conferred by the transmitted Gag sequence is a contributing viral characteristic of that donor virus responsible for influencing early set point VL in the newly infected partner.

### Several amino acids in Gag significantly correlate to changes in replicative capacity

In a pair-wise analysis, a large number of residues were associated with changes in fitness (p<0.05, q<0.51), with 4 residues at 3 unique positions at q<0.2. These residues included the polymorphisms 30R and 31I in p17 (MA), and 309S in p24 (CA). However, in an exploratory analysis of those residues associated with changes in RC with a p value<0.05, it was clear that associated polymorphisms were noticeably enriched on a per residue basis in p17 and p2 (Fisher's exact test, p<0.001). The former plays critical roles in intracellular trafficking, and membrane association of Gag [73], [79], [80], while the latter is an important structural element involved in formation of the immature protein shell [81], [82] and a target of the novel drug Bevirimat during maturational cleavage of the Gag precursor [83], [84]. Surprisingly, only one third of the associated mutations negatively affected virus replication, while nearly two-thirds of the associations increased fitness. Some of these fitness-increasing mutations represent adapted polymorphisms (i.e. selected as immune escape) and in terms of vaccine design it may be important to avoid the inclusion of such epitopes.

Polymorphisms positively or negatively affecting replication in the p24 region of Gag were limited to just six residues (4 positive, 2 negative), in accordance with the conserved nature of this protein. Surprisingly, none of the canonical B*57/B*5801 associated escape mutations within p24, whose fitness defects have been well documented [24], [31]–[34], were found to be significantly associated with decreases in RC in our present study. This may be due to the high prevalence of B*57/B*5801 positive individuals within this cohort (25%), which could promote viral adaptation to these alleles over time through compensatory mutations [22]. It is also possible that some fitness defects such as those associated with T242N within the TW10 epitope might be missed in the current study, as a previous study has shown that it is cell-type dependent [32].

The most deleterious HLA-associated mutation that we observed was K12E, which reduced RC by almost 10-fold relative to the median RC of the cohort. This polymorphism is found quite rarely in the population (3 out of 149), and is statistically associated with HLA-A*74, an allele found to be highly protective in both this Zambian subtype C cohort as well as others [9], [85]. The protective effect of A*74 has recently been demonstrated to be independent of HLA-B*57 [86]. The negative *in vitro* impact of mutations at residue 12 on replication is supported by a longitudinal study of a subset of this seroconvertor cohort (n = 81), in whom polymorphisms at residue 12 were found to revert at a high rate (25%/yr), over the first two years of infection (Schaefer *et al.*, manuscript in preparation). Furthermore, in this same study, escape at position 12 occurred only once and at 24 months post-infection in a total of ten A*74 positive individuals, confirming the high fitness cost associated with CTL-induced escape mutations at this position. We hypothesize that the targeting of this putative epitope, KR9 [86], may account for part of the protective effect conferred by A*74 and indicates that protective immune responses can target regions of Gag outside of p24. While the nature of the replication defect in viruses encoding K12E remains to be determined, this residue does lie in the highly basic region at the N-terminus of p17 (MA), which is involved in membrane targeting and membrane association of Gag [80], [87], [88].

### Rare polymorphisms have the greatest effect on replicative capacity

Rare mutations, such as K12E, which occur in a small subset of the population studied here (less than 10 individuals of the 149), affected fitness to a statistically greater degree than more common polymorphisms. Rare fitness decreasing mutations are likely unique to specific circumstances such as those where a considerable decrease in RC is warranted in the face of a very effective cellular immune response that is largely abrogated upon mutation. Such mutations have been found to subsequently revert after transmission to individuals lacking the selecting HLA-allele [17], [42], [89] and in whom they now confer a fitness deficit for the virus. These sites of rare fitness reducing polymorphisms may emphasize vulnerable epitopes at which HIV-1 escapes from immune pressure with great difficulty. Alternatively, it is possible that, when escape occurs, it is consistently associated with a decrease in RC that cannot be completely compensated.

A similar observation was made for rare mutations that greatly increase RC. Global compensatory mutations do exist that can compensate multiple deleterious mutations, such as those within the cyclophilin binding loop [42], [46]. Some of the rare fitness increasing mutations may be of this type, although those reported previously have generally been quite common in the population. Compensatory mutations can also be secondary site-suppressors of deleterious mutations [90]. Frequently, such mutations are only conditionally beneficial and can be deleterious in a different context, which could explain why some fitness increasing mutations are rare. It is also possible that these mutations do carry some unrecognized *in vivo* fitness cost that cannot be captured in the *in vitro* replication system used here. Due to the fact that these mutations are rare, they are difficult to statistically link to HLA alleles or to link to other residues with which they may covary, making the potential fitness defects that these mutations mitigate difficult to elucidate.

### The cumulative and qualitative effect of HLA associated polymorphisms in Gag on replicative capacity and VL

A key goal of this study was to understand how the cellular immune response might select for mutations in Gag that reduce viral RC, and while identification of specific amino acid polymorphisms that either increase or decrease fitness can be informative, it is equally important to elucidate how the accumulation of specific HLA-associated polymorphisms in Gag affects both RC of the virus and VL in the newly infected person. Previous efforts to correlate the total number of HLA-associated polymorphisms in Gag to RC have yielded inconclusive results [32], [54], perhaps because the quality of the polymorphisms in question was not considered. Using an expanded list of HLA-associated polymorphisms (Carlson, Schaefer *et al.*, manuscript in preparation) we report a weak positive correlation between the total number of HLA-associated polymorphisms in Gag and RC. The fact that this correlation was positive is consistent with our observation that a large fraction of the non-consensus HLA-associated polymorphisms increased RC. In particular, in the expanded data set of HLA-associated polymorphisms, we observed that non-adapted residues, which would be predicted to render the virus susceptible to the linked HLA allele, were statistically associated with increased fitness.

These findings suggest that CTL escape mutations, which decrease the overall RC of the virus, are being driven to consensus as a result of population level immune pressure. In the absence of immune pressure, the non-escaped (non-adapted) residues would be expected to predominate, but if they render the virus susceptible to a large portion of the population, then the consensus residue will be escaped rather than susceptible, despite reducing *in vitro* fitness. This is consistent with the findings of Kawashima *et al.* [22] that the frequency of certain HLA-class I alleles within a particular population can influence the fixation of escape mutations in the overall population. Moreover, Wright *et al.* [55] showed that Gag-NL43 recombinant viruses encoding *gag-pro* sequences most disparate from the subtype C consensus *gag-pro* sequence had statistically higher replicative capacities than their more consensus-like counterparts, and this finding has been recapitulated in this current study. Taken together, these data suggest that overall, HLA-mediated adaptation is driving the fixation of consensus residues that are less fit than their susceptible counter-parts.

When we account for this ability of HLA-associated polymorphisms to either increase or decrease fitness by assigning a summed polymorphism score, which subtracts the number of fitness decreasing polymorphisms from the number of fitness increasing polymorphisms in a particular sequence, we find a highly statistically significant correlation between RC and the summed polymorphism score (p<0.0001). Although this p-value should be interpreted cautiously, since it reflects the summation of features previously identified to be correlated with RC, the data do suggest that the effect of polymorphisms is cumulative, and that as a Gag sequence accumulates an excess of fitness-reducing polymorphisms, the RC decreases proportionally. Similarly, utilization of a summed polymorphism score improved previously reported correlations between the total number of HLA-associated polymorphisms in Gag and set point VL in newly infected individuals [50]. We observed a highly statistically significantly correlation (p = 0.006) between the summed polymorphism score and set point VL in newly infected individuals. Just as this balance of fitness increasing and decreasing polymorphisms impacts RC, it simultaneously influences the set point VL of the newly infected individual.

### Transmission of viruses with low RCs provides recipients with a longer-term clinical benefit

While VL has been demonstrated to influence the rate of disease progression in HIV-1 infected individuals [68], [69], it is possible that, during the very earliest stages of infection and before host immune control, the replication rate of the virus may affect the rate of future damage to the immune system. Indeed, we observed a statistically significant negative correlation between RC and average CD4 counts for the first year post infection, suggesting a role for RC in defining this important parameter of pathogenesis at early stages after infection. However, it is possible that this early benefit could be quickly lost due to further adaptation of the virus to the new host's immunogenetic background and further compensation for *de novo* escape mutations. Consequently, we analyzed individuals with longitudinal CD4 counts out to three-years post infection in order to determine if the observed early benefit was sustained in early chronic stages of infection. Using Kaplan-Meier survival analyses to examine the relative time for individuals infected with viruses encoding *gag* genes conferring RC values of <1 and >2 to reach CD4 T cell counts of 350 after 3 years of infection, we observed a clear and statistically significant difference. This was even more striking when CD4 counts less than 300 were used as the endpoint. Moreover a Cox proportional hazard model demonstrated a significantly increased risk of CD4 counts falling below both 350 (HR 2.36) or 300 (HR 3.80) over the first three years of infection for individuals whose *gag* gene conferred an RC>2 vs. RC<1. These findings indicate that the RC conferred by the transmitted Gag sequence may have profound and prolonged effects on HIV-1 pathogenesis from acute to early chronic stages of infection.

While RC and VL are correlated in the full data set, set point VL does not fully explain the effect of RC on CD4, because we did not observe any statistically significant differences in set point VL between the two groups (RC<1 and RC>2) for the subset of individuals with CD4+T cell counts (n = 63). Moreover, in Cox proportional hazard models which take into account VL, the HR remained high (2.17 and 3.11 respectively) and p values continued to trend or remain borderline significant (p = 0.093 and 0.051). This suggests that both VL and RC have independent effects on CD4 decline, however, because this analysis was conducted on a subset of less than half of our initial cohort, additional work is underway to further confirm and extend these results.

It seems possible, therefore, that the RC of the transmitted variant may initiate crucial events, early in infection and dissemination, that dictate both acute and later stage pathogenesis regardless of the ability of the immune system to control viral replication down to set point. Infection with highly replicating variants could lead to a more complete depletion of central memory CD4+ T cell pools at this early time that could predispose an individual to more rapid CD4+ T cell loss, irrespective of adequate control of viral replication. This is evidenced in a few individuals infected with highly replicating Gag variants (RC>2), who go on to control VL to a low set point, but whose CD4+ T cells counts rapidly drop below 300 (data not shown). Additionally, a high level of peak viremia or initial high antigen loads could establish an inflammatory environment that leads to sustained immune activation, which has been implicated as a more reliable marker for disease progression [91]. These possibilities are the focus of ongoing work.

In summary, using an *in vitro* approach to define the impact of polymorphisms in Gag on transmitted virus RC has clearly shown that this property of the virus is a significant contributor to early set point VL in a newly infected individual. More importantly, however, these studies suggest a critical role for RC in defining the trajectory of immune depletion and pathogenesis, beyond simply its impact on VL, and highlight the importance of the very earliest events in virus-host interactions. It also raises the possibility that a vaccine that can attenuate early virus replication would have a positive impact both on vaccinated individuals, as well as non-vaccinated individuals by weakening the transmitted/founder virus and increasing the likelihood of transmission of low replicating variants.

## Supporting Information

Figure S1 **Donor and recipient population** ***gag*** **sequences cluster with one another.** Gag population sequences from donors and linked recipients were amplified and sequenced as described in the methods section. Nucleotide *gag* sequences were aligned using the Gene Cutter tool accessible on the Los Alamos Nation Lab HIV Sequence Database (http://www.hiv.lanl.gov/content/sequence/GENE_CUTTER/cutter.html) and a neighbor-joining tree was generated using the Geneious sequence analysis software v5.5.7 (Biomatters Ltd.). The radial tree was annotated using the Interactive Tree of Life (iTOL) online tool for phylogenetic tree display and annotation (Letunic and Bork, Bioinformatics, 2006). Blue denotes transmitting partners (donors) and green denotes linked recipients. This tree demonstrates the high degree of similarity between donor and linked recipient *gag* population sequences within epidemiologically linked transmission pairs.
(EPS)

Figure S2 **Gag sequences that are less like the Gag subtype C consensus sequence replicate more efficiently** ***in vitro*** **.** The Gag amino acid sequences of all Gag-MJ4 chimeras were compared to a Zambian subtype C consensus Gag sequence (generated using the LANL Consensus Maker tool; http://www.hiv.lanl.gov/content/sequence/CONSENSUS/consensus.html) by building a neighbor-joining tree using the Geneious sequence analysis software v5.5.7 (Biomatters Ltd.) and the percent similarity to consensus was determined for each sequence. Notably, viruses encoding Gag sequences most disparate from the subtype C consensus Gag sequence replicated to higher levels, and we observed a statistically significant negative correlation between RC and the percent similarity of Gag to consensus (Pearson correlation, p = 0.002, r = −0.23).
(EPS)

Table S1 **Amino acids in Gag associated with changes in replicative capacity.** This table lists all amino acids associated with changes in RC. Residues that remain significantly associated with changes in RC after correction for multiple comparisons (q<0.2) are depicted in green. A total of 152 sequences and RC values were available for association analysis, with 149 of these with sufficient clinical follow-up for inclusion in the broader study. *^a^* ΔRC is defined as median RC of all viruses tested (∼1.5) subtracted from the median RC of all viruses with the particular polymorphism. *^b^* The location of epitopes was defined by the compendium of “A-list” epitopes available in the LANL Immunology Database (*HIV Molecular Immunology 2009*). *^c^* HLA class I alleles restricting epitopes harboring these polymorphisms that affect RC were also defined base on the LANL Immunology Database compilation of “A-list” epitopes.
(DOC)
