# Supplementary material for: Role of Transmitted Gag CTL Polymorphisms in Defining Replicative Capacity and Early HIV-1 Pathogenesis
Source: PLoS Pathog. 2012 Nov 29;8(11):e1003041. doi: 10.1371/journal.ppat.1003041 (PMC3510241; doi:10.1371/journal.ppat.1003041)
Supplement: Table S1 — Amino acids in Gag associated with changes in replicative capacity. This table lists all amino acids associated with changes in RC. Residues that remain significantly associated with changes in RC after correction for multiple comparisons (q<0.2) are depicted in green. A total of 152 sequences and RC values were available for association analysis, with 149 of these with sufficient clinical follow-up for inclusion in the broader study. a ΔRC is defined as median RC of all viruses tested (∼1.5) subtracted from the median RC of all viruses with the particular polymorphism. b The location of epitopes was defined by the compendium of “A-list” epitopes available in the LANL Immunology Database (HIV Molecular Immunology 2009). c HLA class I alleles restricting epitopes harboring these polymorphisms that affect RC were also defined base on the LANL Immunology Database compilation of “A-list” epitopes. (DOC) [file ppat.1003041.s003.doc]

| Codon | Amino Acid | Consensus Amino Acid | Sequences w/o residue | Sequences with residue | Median RC w/o residue | Median RC with residue | p value | q value | ΔRC*^a^* | Within Epitope*^b^* | Epitope HLA Restriction*^c^* |
| --- | --- | --- | --- | --- | --- | --- | --- | --- | --- | --- | --- |
| 31 | L | L | 49 | 103 | 1.051613 | 1.694342 | 0.00001 | 0.00545 | 0.19326 | YES | A*2402; B*0801 |
| 31 | I | L | 119 | 33 | 1.606183 | 0.974471 | 0.00016 | 0.04360 | -0.52661 | YES | A*2402; B*0801 |
| 30 | R | M | 130 | 22 | 1.442259 | 2.080627 | 0.00107 | 0.18394 | 0.57955 | YES | A*2402; B*0801 |
| 309 | S | A | 126 | 26 | 1.572069 | 0.890472 | 0.00135 | 0.18394 | -0.61061 | YES | B*4402; B*5301; B*5701; Cw5 |
| 28 | R | H | 125 | 27 | 1.433357 | 1.974499 | 0.00326 | 0.32077 | 0.47342 | YES | A*0301; A*2402; B*0801 |
| 309 | A | A | 29 | 123 | 0.910234 | 1.567691 | 0.00397 | 0.32077 | 0.06661 | YES | B*4402; B*5301; B*5701; Cw5 |
| 370 | A | A | 48 | 104 | 1.797895 | 1.380252 | 0.00412 | 0.32077 | -0.12083 | YES | B*4501 |
| 4 | S | R | 149 | 3 | 1.484227 | 2.651962 | 0.00965 | 0.40790 | 1.15088 | NO |  |
| 4 | R | R | 3 | 149 | 2.651962 | 1.484227 | 0.01005 | 0.40790 | -0.01685 | NO |  |
| 451 | N | S | 96 | 56 | 1.417936 | 1.713877 | 0.01179 | 0.40790 | 0.21280 | NO |  |
| 111 | S | S | 61 | 90 | 1.74016 | 1.383489 | 0.01199 | 0.40790 | -0.11759 | NO |  |
| 418 | K | K | 22 | 130 | 2.0575 | 1.468355 | 0.01217 | 0.40790 | -0.03272 | NO |  |
| 373 | A | N | 125 | 4 | 1.534145 | 0.764576 | 0.01258 | 0.40790 | -0.73650 | YES | B*4501 |
| 146 | S | A | 142 | 10 | 1.468355 | 2.255673 | 0.01380 | 0.40790 | 0.75460 | YES | A*2501; B*1510; B*5701 |
| 119 | A | E | 145 | 4 | 1.472093 | 2.387669 | 0.01388 | 0.40790 | 0.88659 | NO |  |
| 121 | A | D | 137 | 15 | 1.472093 | 2.244058 | 0.01418 | 0.40790 | 0.74298 | NO |  |
| 42 | D | E | 145 | 7 | 1.534145 | 0.769036 | 0.01449 | 0.40790 | -0.73204 | YES | A*30; B*3501 |
| 451 | S | S | 63 | 89 | 1.68293 | 1.386771 | 0.01499 | 0.40790 | -0.11431 | NO |  |
| 69 | Q | Q | 26 | 126 | 1.822397 | 1.442259 | 0.01663 | 0.40790 | -0.05882 | NO |  |
| 12 | E | K | 149 | 3 | 1.532977 | 0.750097 | 0.01901 | 0.40790 | -0.75098 | YES | B*4002 |
| 418 | R | K | 136 | 16 | 1.47816 | 2.245238 | 0.01981 | 0.40790 | 0.74416 | NO |  |
| 319 | E | D | 125 | 27 | 1.464616 | 1.735435 | 0.02014 | 0.40790 | 0.23436 | NO |  |
| 319 | D | D | 27 | 125 | 1.735435 | 1.464616 | 0.02014 | 0.40790 | -0.03646 | NO |  |
| 85 | L | L | 4 | 148 | 2.364864 | 1.47816 | 0.02077 | 0.40790 | -0.02292 | YES | A*0201; A*1101; A*2902; A*3002; B*4403; B*58; B*63; Cw14 |
| 111 | C | S | 110 | 41 | 1.417936 | 1.819739 | 0.02115 | 0.40790 | 0.31866 | NO |  |
| 480 | G | D | 145 | 3 | 1.49407 | 2.632309 | 0.02118 | 0.40790 | 1.13123 | NO |  |
| 62 | K | K | 61 | 91 | 1.68293 | 1.386563 | 0.02145 | 0.40790 | -0.11451 | NO |  |
| 312 | E | D | 79 | 73 | 1.588149 | 1.451162 | 0.02178 | 0.40790 | -0.04992 | YES | B*4402; B*5301; B*5701; Cw5 |
| 312 | D | D | 73 | 79 | 1.451162 | 1.588149 | 0.02178 | 0.40790 | 0.08707 | YES | B*4402; B*5301; B*5701; Cw5 |
| 123 | E | G | 133 | 13 | 1.535189 | 1.002543 | 0.02266 | 0.40790 | -0.49853 | NO |  |
| 495 | N | S | 117 | 35 | 1.451162 | 1.952761 | 0.02395 | 0.40790 | 0.45168 | NO |  |
| 495 | S | S | 35 | 117 | 1.952761 | 1.451162 | 0.02395 | 0.40790 | -0.04992 | NO |  |
| 228 | L | M | 141 | 11 | 1.472093 | 2.273388 | 0.02650 | 0.43765 | 0.77231 | YES | B*13 |
| 103 | K | K | 10 | 142 | 2.086015 | 1.474422 | 0.03202 | 0.48755 | -0.02666 | NO |  |
| 371 | S | N | 148 | 3 | 1.520532 | 0.778439 | 0.03300 | 0.48755 | -0.72264 | YES | B*4501 |
| 67 | S | A | 145 | 7 | 1.472093 | 2.139988 | 0.03310 | 0.48755 | 0.63891 | NO |  |
| 67 | A | A | 7 | 145 | 2.139988 | 1.472093 | 0.03310 | 0.48755 | -0.02898 | NO |  |
| 103 | R | K | 143 | 9 | 1.484227 | 2.139988 | 0.03564 | 0.50140 | 0.63891 | NO |  |
| 373 | Q | N | 124 | 5 | 1.47816 | 2.15603 | 0.03588 | 0.50140 | 0.65495 | YES | B*4501 |
| 42 | E | E | 10 | 142 | 0.903087 | 1.534667 | 0.03691 | 0.50290 | 0.03359 | YES | A*30; B*3501 |
| 121 | G | D | 135 | 17 | 1.538821 | 1.099119 | 0.03823 | 0.50552 | -0.40196 | NO |  |
| 76 | K | R | 110 | 42 | 1.394643 | 1.714888 | 0.04012 | 0.50552 | 0.21381 | YES | A*3002; B*0801; B*4403; B*58; B*63 |
| 28 | T | H | 148 | 4 | 1.533561 | 0.716807 | 0.04064 | 0.50552 | -0.78427 | YES | A*0301; A*2402; B*0801 |
| 488 | A | S | 141 | 11 | 1.472093 | 2.013763 | 0.04174 | 0.50552 | 0.51269 | YES | B*4001 |
| 488 | S | S | 11 | 141 | 2.013763 | 1.472093 | 0.04174 | 0.50552 | -0.02898 | YES | B*4001 |
| 479 | R | K | 128 | 22 | 1.459385 | 1.733306 | 0.04370 | 0.51775 | 0.23223 | NO |  |
| 31 | M | L | 136 | 16 | 1.534083 | 1.109909 | 0.04710 | 0.54616 | -0.39117 | YES | A*2402; B*0801 |
| 256 | V | I | 99 | 53 | 1.454154 | 1.606183 | 0.04825 | 0.54784 | 0.10511 | YES | B*3501 |
| 374 | V | T | 149 | 3 | 1.484227 | 2.642477 | 0.04981 | 0.55401 | 1.14140 | YES | B*4501 |

This table lists all amino acids associated with changes in RC. Residues that remain significantly associated with changes in RC after correction for multiple comparisons (q<0.2) are depicted in green. A total of 152 sequences and RC values were available for association analysis, with 149 of these with sufficient clinical follow-up for inclusion in the broader study.

*^a^* ΔRC is defined as median RC of all viruses tested (~1.5) subtracted from the median RC of all viruses with the particular polymorphism.

*^b^* The location of epitopes was defined by the compendium of “A-list” epitopes available in the LANL Immunology Database (**HIV Molecular Immunology 2009).**

**^c^ HLA class I alleles restricting epitopes harboring these polymorphisms that affect RC were also defined base on the LANL Immunology Database compilation of “A-list” epitopes.**
